# Tau-PET and CSF MTBR-tau243 comparisons validate increased tau aggregation in females

**DOI:** 10.1007/s00259-026-07934-y

**Published:** 2026-05-27

**Authors:** Carling G. Robinson, Alexa Pichet Binette, Kanta Horie, Chihiro Sato, Suzanne E. Schindler, Randall J. Bateman, Tammie L. S. Benzinger, Shorena Janelidze, Ellen Singleton, Erik Stomrud, Gordon Zhaoqi An, Taylor J. Pedersen, Sebastian Palmqvist, Niklas Mattsson-Carlgren, John C. Morris, Oskar Hansson, Brian A. Gordon, Rik Ossenkoppele

**Affiliations:** 1 Department of Psychological and Brain Sciences, Washington University in St. Louis, St. Louis, MO USA; 2 Department of Radiology, Washington University in St. Louis, St. Louis, MO USA; 3 Department of Physiology and Pharmacology, Université de Montréal, Montréal, QC Canada; 4 Canada Centre de Recherche de L’Institut Universitaire de Gériatrie de Montréal, Montréal, QC Canada; 5 Clinical Memory Research Unit, Department of Clinical Sciences Malmö, Lund University, Lund, Sweden; 6 Department of Neurology, Washington University in St. Louis, St. Louis, MO USA; 7 Tracy Family SILQ Center, Washington University School of Medicine, St. Louis, MO USA; 8 Eisai Inc, Nutley, NJ USA; 9 Alzheimer Center Amsterdam, Department of Neurology, Vrije Universiteit Amsterdam, Amsterdam UMC VUmc, Amsterdam, The Netherlands; 10 Department of Neurodegeneration, Amsterdam Neuroscience, Amsterdam, The Netherlands; 11 Wallenberg Center for Molecular Medicine, Lund University, Lund, Sweden

**Keywords:** Sex, Females, Tau, Alzheimer’s disease (AD), PET, MTBR-tau243

## Abstract

**Background:**

Females consistently demonstrate greater tau Positron Emission Tomography (PET) tracer signal. Although interpreted as reflecting greater Alzheimer disease (AD) pathology, it’s unclear whether differences arise from biological mechanisms or methodological factors. Microtubule binding region tau species containing residue 243 (MTBR-tau243) in cerebrospinal fluid (CSF) is a biomarker of aggregated tau but avoids PET limitations such as off-target binding. Comparing tau-PET and CSF MTBR-tau243, including sex interactions, can help elucidate the origin of sex differences in tau.

**Purpose:**

Conduct a cross-sectional analysis of CSF MTBR-tau243 and tau-PET by sex in the Swedish BioFINDER-2 (BioFINDER-2) Study and Charles F. and Joanne Knight Alzheimer Disease Research Center (Knight-ADRC).

**Methods:**

Participants had CSF MTBR-tau243, tau-PET, and Aβ status defined by the CSF Aβ42/40 ratio. Tau-PET was measured using [^18^F]Flortaucipir (Knight-ADRC, *N* = 219) and [^18^F]RO948 (BioFINDER-2, *N* = 446). We tested the interaction between sex and CSF MTBR-tau243 on tau-PET burden in a temporal meta region-of-interest (ROI) across all participants and in Aβ-positive participants.

**Results:**

In both cohorts, CSF MTBR-tau243 was associated with tau-PET temporal meta-ROI burden (BioFINDER-2: β = 1.1, *p* = < 0.0001 and Knight-ADRC: β = 0.7, *p* = < 0.0001), and this association did not differ by sex (BioFINDER-2 β = 0.04, *p* = 0.6 and Knight-ADRC: β = 0.009, *p* = 0.9). Among Aβ-positive subgroups, results remained consistent for the main effect (BioFINDER-2: β = 1.1, *p* = < 0.0001 and Knight-ADRC: β = 0.7, *p* = < 0.0001) and interaction (BioFINDER-2: β = 0.04, *p* = 0.7 and Knight-ADRC: β = 0.06, *p* = 0.6).

**Conclusion:**

The lack of sex moderating the association between tau-PET and CSF MTBR-tau243 indicates that higher tau-PET signal in females reflects greater susceptibility to tau pathology rather than a methodological artifact.

**Supplementary Information:**

The online version contains supplementary material available at 10.1007/s00259-026-07934-y.

## Introduction

Understanding heterogeneities in tau burden, including the role of biological sex in shaping susceptibility and propagation, is critical for informing the development and optimization of therapeutic interventions for Alzheimer disease (AD). Females are disproportionately affected by AD [1, 2], accounting for approximately two-thirds of cases [3]. This sex difference in prevalence is consistently observed and cannot be solely explained by females’ longer life expectancy [1, 4]. 

AD pathophysiology follows a well-characterized sequence in which amyloid-beta (Aβ) accumulation initiates downstream tau accumulation, neurodegeneration, and cognitive impairment [5, 6]. Compared to Aβ, tau is more closely associated with neurodegeneration and cognitive decline [7]. Although males and females exhibit similar levels of Aβ pathology [8–10], females exhibit greater tau measured with positron emission tomography (PET) [11–18], and at autopsy [19–23]. Importantly, sex-specific vulnerability to tau is pronounced with elevated Aβ [12, 15–17], with research showing that for a given Aβ burden, females accumulate tau more rapidly than males [11, 12]. Collectively, these findings suggest that females are more vulnerable to tau pathology.

The importance of understanding the interaction between tau pathology and sex has potential therapeutic implications. First, recently approved amyloid targeting therapies [24, 25] demonstrated a modest overall cognitive benefit, which was most pronounced in individuals in earlier clinical and biological disease stages. Some clinical trials have also suggested reduced therapeutic efficacy in females, although sex interactions were not formally tested [26]. This represents an important question that should be considered in future clinical trials. Finally, sex-specific differences in susceptibility to tau pathology may be particularly relevant for the development of effective tau targeting therapies.

Despite many strengths of PET, important limitations remain. Tau-PET tracers have high specificity for tau aggregates, but off-target retention is documented in the basal ganglia, skull, choroid plexus, and meninges [27, 28]. Although often interpreted as a potentiation of AD pathology in females, tau-PET sex differences may partly reflect sex-related variation in off-target binding [11, 29–31]. Establishing whether females truly exhibit higher tau pathology, rather than an artifact of tau-PET imaging, is therefore critical for accurately interpreting treatment response patterns and for informing the development of effective therapeutic strategies, particularly tau-targeted therapies [32]. Microtubule binding region tau species containing residue 243 (MTBR-tau243) in cerebrospinal fluid (CSF) provides a measure of aggregated tau comparable to tau-PET [33, 34] but bypasses PET-related methodological limitations. MTBR-tau243, therefore, provides a means to disentangle the biological versus methodological contributions to observed sex differences in tau-PET.

The present study examined the moderating effect of sex on the relationship between CSF MTBR-tau243 and tau-PET, using cross-sectional data from two independent cohorts. Since both tau-PET and MTBR-tau243 measure aggregated tau, such that sex effects on tau pathology would be expected to manifest similarly across these biomarkers, we hypothesized that sex would not moderate the association between CSF MTBR-tau243 and tau-PET.

## Materials and methods

### Participant inclusion criteria

Participants were selected from the Swedish BioFINDER-2 (BioFINDER-2) Study (NCT03174938) at Lund University and the Charles F. and Joanne Knight Alzheimer Disease Research Center (Knight-ADRC) at Washington University in St. Louis (WashU). BioFINDER-2 participants were recruited in southern Sweden from 2017 to 2021. Knight-ADRC participants were recruited in St. Louis, MO, and surrounding cities from 2014 to 2020. Participants were required to have baseline data for CSF MTBR-tau243, tau-PET, and Aβ status defined by the CSF Aβ42/40 ratio. 665 participants met inclusion criteria (Knight-ADRC *n* = 219; BioFINDER-2 *n* = 446). Sex was self-reported and coded in a binary fashion (male/female). Race and ethnicity were self-reported in Knight-ADRC but not available in BioFINDER-2, where the majority is White. This study followed the Strengthening the Reporting of Observational Studies in Epidemiology (STROBE) reporting guidelines [35].

### Neuroimaging analysis

Tau-PET was measured using [^18^F]RO948 in BioFINDER-2 [36] and [^18^F]Flortaucipir in the Knight-ADRC [37]. The primary tau-PET region of interest (ROI) was the temporal meta-ROI, defined as the weighted mean standardized uptake value ratio (SUVr) of the entorhinal cortex, amygdala, inferior/middle temporal gyri, fusiform gyrus, and parahippocampal gyrus [38–42]. SUVrs were generated using the inferior cerebellum as the reference region in BioFINDER-2 and the cerebellar grey in the Knight-ADRC.

### CSF markers

Aβ status was determined as a binary variable using the Elecsys immunoassays (Roche Diagnostics) CSF Aβ42/40 ratio in BioFINDER-2 [43] and the LUMIPULSE CSF Aβ42/40 ratio in the Knight-ADRC [44]. In BioFINDER-2, a pre-established cutoff of < 0.08 [43] ratio was used to define Aβ-positivity. A few participants did not have Elecsys measurements available, in which case clinical routine assays and pre-established cutoffs were used [43]. In the Knight-ADRC, Aβ-positive participants were selected based on a pre-established cutoff of < 0.0673 [34, 44–47]. Measurement of CSF tau species MTBR-tau243 was performed at WashU in both cohorts using immunoprecipitation/mass spectrometry as previously described [34, 45].

### Clinical assessment

In both cohorts, the Mini-Mental State Examination (MMSE) [48] quantified impairment severity. The Knight-ADRC also utilized Clinical Dementia Rating® (CDR) scores [49]. When judged to have impairments, a clinical diagnosis indicating the cause of impairment was provided by clinicians.

### Statistical analyses

Linear regression models evaluated the association between baseline CSF MTBR-tau243 and baseline tau-PET uptake. To assess sex differences, models evaluated the potential moderating effect of sex on the association between baseline CSF MTBR-tau243 and baseline tau-PET. The main analysis of interest was whether there was a significant interaction between sex and baseline CSF MTBR-tau243 in predicting tau-PET uptake within a temporal meta-ROI. To examine this relationship in the context of elevated Aβ pathology, analyses were repeated only in Aβ-positive participants (Aβ-positive BioFINDER-2: *n* = 302, Aβ-positive Knight ADRC: *n* = 135). All analyses included age, sex, and Aβ status as covariates (except for analyses in Aβ-positive subgroups). In sensitivity analyses, we tested the same interaction with tau-PET uptake in the entorhinal cortex (early stage) as the outcome. Supplementary analyses were also performed in each of the Freesurfer tau-PET ROIs, and whole-brain maps were generated to visualize potential sex effects. We accounted for multiple comparisons using the Benjamini–Hochberg false discovery rate correction (FDR; q < 0.05). Finally, we report sex-stratified slope estimates for the association between MTBR-tau243 and tau-PET uptake derived from the existing regression models in the BioFINDER-2 and Knight-ADRC cohorts, including the Aβ-positive subgroups. All available data were included in analyses; no observations were excluded as outliers. Analyses were performed using R version 2025.05.0 + 496.

## Results

**Fig. 1:**
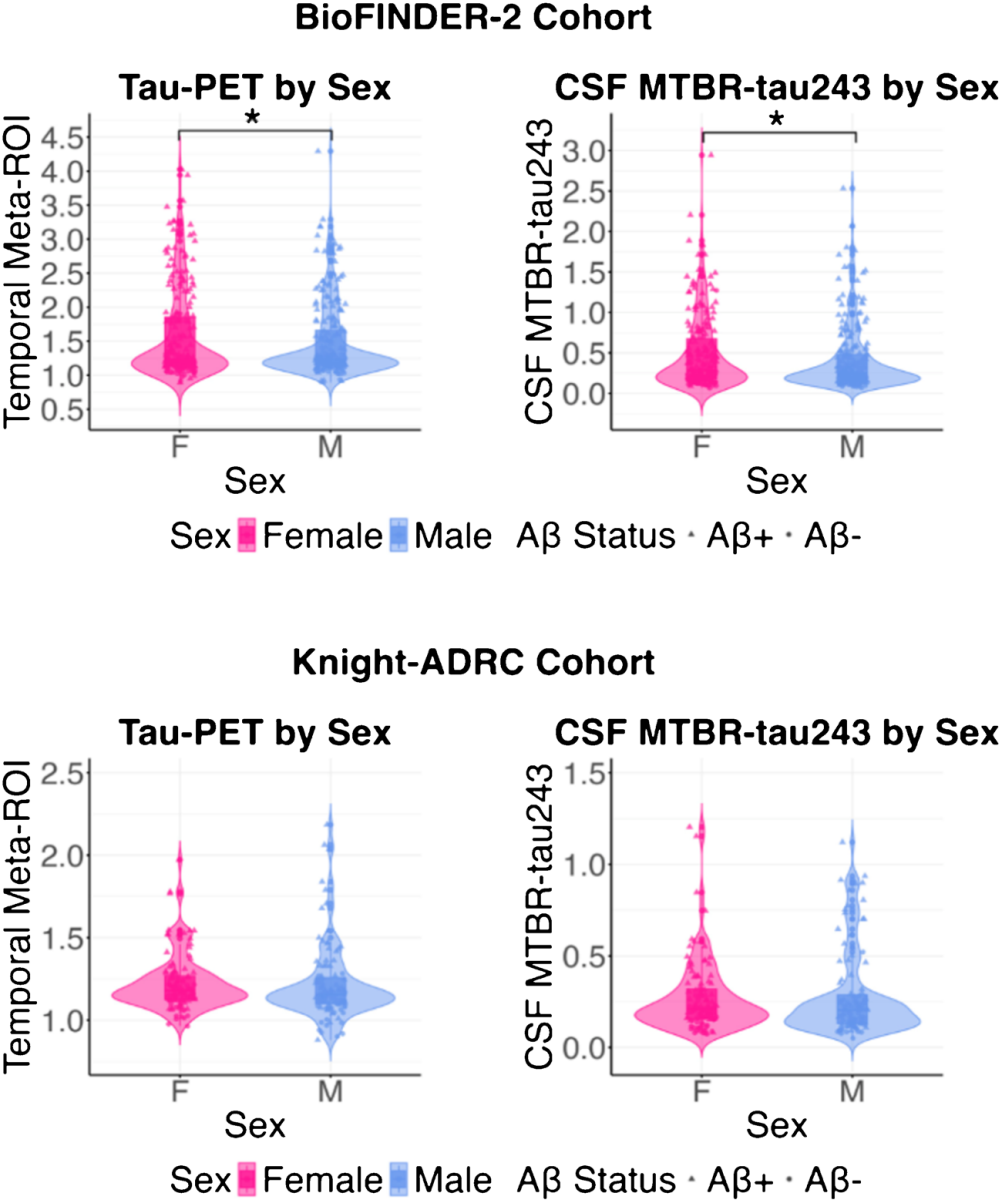
Comparisons of tau pathology measured by tau-PET and CSF MTBR-tau243 by sex. Knight Alzheimer Disease Research Center (Knight-ADRC), positron emission tomography (PET), cerebrospinal fluid (CSF), microtubule binding region tau species containing residue 243 (MTBR-tau243), amyloid-beta (Aβ). The tau-PET temporal meta-ROI encompassed regions including the entorhinal cortex, amygdala, inferior/middle temporal gyri, fusiform gyrus, and parahippocampal gyrus. Females shown in pink, males shown in blue. *Denotes statistical significance (*p* < 0.05). In BioFINDER-2, females had higher CSF MTBR-tau243 levels (β = 0.08, *p* = 0.03) and tau-PET burden in the temporal meta-ROI (β = 0.1, *p* = 0.04) than males. In the Knight-ADRC no sex differences were observed in CSF MTBR-tau243 levels (β = −0.03, *p* = 0.3) or tau-PET burden (β = −0.004, *p* = 0.9)

**Fig. 2:**
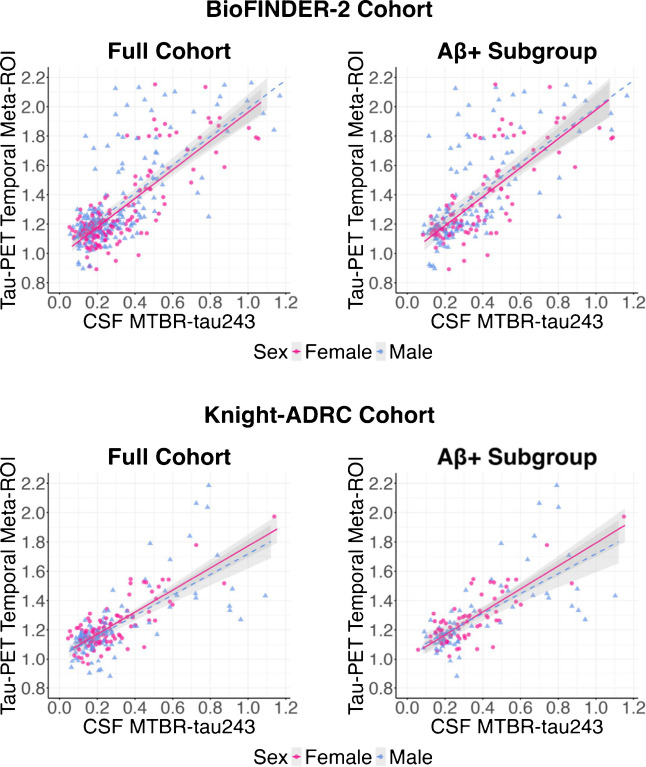
Associations between Tau-PET and CSF MTBR-tau243. Knight Alzheimer Disease Research Center (Knight-ADRC), positron emission tomography (PET), cerebrospinal fluid (CSF), microtubule binding region tau species containing residue 243 (MTBR-tau243), amyloid-beta (Aβ). The tau-PET temporal meta-ROI encompassed regions including the entorhinal cortex, amygdala, inferior/middle temporal gyri, fusiform gyrus, and parahippocampal gyrus. Females shown in pink, males shown in blue. No significant sex-by-MTBR-tau243 interaction was observed in the full BioFINDER-2 cohort (β = 0.04, *p* = 0.6) or the full Knight-ADRC cohort (β = 0.009, *p* = 0.9), nor in the Aβ-positive BioFINDER-2 subgroup (β = 0.04, *p* = 0.7) or the Aβ-positive Knight-ADRC subgroup (β = 0.06, *p* = 0.6)

Demographic data for the BioFINDER-2 (*n* = 446) and the Knight ADRC (*n* = 219) cohorts are in Table 1. The proportion of female participants was comparable across cohorts, comprising 48.9% of BioFINDER-2 (*n* = 218) and 51.1% of the Knight-ADRC (*n* = 112). In both cohorts, the association between APOE ε4 carrier status and sex was not significant (BioFINDER-2: 129 males vs 128 females; χ^2^ = 0.2, *p* = 0.7; Knight-ADRC: 45 males vs 51 females; χ^2^ = 0.3, *p* = 0.9). No significant sex differences in age were observed. In BioFINDER-2, females had higher CSF MTBR-tau243 levels (β = 0.08, *p* = 0.03) and tau-PET burden in the temporal meta-ROI (β = 0.1, *p* = 0.04) than males (Fig. 1), while there were no sex differences on these two measures in the Knight-ADRC (Tau-PET: β = −0.004, *p* = 0.9 and CSF MTBR-tau243: β = −0.03, *p* = 0.3). In both cohorts, there were significant associations between CSF MTBR-tau243 and the tau-PET temporal meta-ROI (BioFINDER-2: β = 1.1, *p* < 0.0001, Knight-ADRC: β = 0.7, *p* < 0.0001). This association was also present in Aβ-positive subgroups of both cohorts (BioFINDER-2: β = 1.1, *p* = < 0.0001, Knight-ADRC β = 0.7, *p* < 0.0001). The central analysis assessed the interaction between CSF MTBR-tau243 and sex on tau-PET burden. In both cohorts, the interaction was neither significant in the whole group (BioFINDER-2: β = 0.04, *p* = 0.6, Knight-ADRC: β = 0.009, *p* = 0.9) (Fig. 2) nor in Aβ-positive subgroups (BioFINDER-2: β = 0.04, *p* = 0.7, Knight-ADRC: β = 0.06, *p* = 0.6) (Fig. 2)*.* In sensitivity analyses, we tested the same interaction with tau-PET uptake in the entorhinal cortex as the outcome, and again, CSF MTBR-tau43 levels and tau-PET burden did not differ by sex (Table 2).

**Table 1:** Demographics of the BioFINDER-2 and Knight-ADRC cohorts

BioFINDER-2			
Variable of Interest	Males	Females	*P*-value
Sex %	51.1% (*n* = 228)	48.9% (*n* = 218)	0.7
Age	71.9 (8.4)	70.9 (8.6)	0.2
Education	12.4 (4.0)	12.0 (3.4)	0.4
*APOE* ε4 carrier %	56.6% (*n* = 129)	58.7% (*n* = 128)	0.7
MMSE score	25.9 (4.5)	25.6 (4.7)	0.6
Clinical diagnosis			0.6
Cognitively Unimpaired %	35.5% (*n* = 81)	35.1% (*n* = 80)	
Mild Cognitive Impairment %	24.6% (*n* = 56)	17.5% (*n* = 40)	
Clinical diagnosis of AD %	20.2% (*n* = 46)	24.6% (*n* = 56)	
Non-AD neurodegenerative disease %	19.7% (*n* = 45)	18.4% (*n* = 42)	
Aβ-positive %	68.0% (*n* = 155)	67.4% (*n* = 147)	0.9
Knight-ADRC			
Sex %	48.9% (*n* = 107)	51.1% (*n* = 112)	0.7
Race			0.7
White%	93.5% (*n* = 100)	94.6% (*n* = 106)	
Black%	4.7% (*n* = 5)	3.6% (*n* = 4)	
Asian%	0.9% (*n* = 1)	1.8% (*n* = 2)	
Mixed Race%	0.9% (*n* = 1)	0% (*n* = 0)	
Age	70.6 (11.0)	70.8 (7.6)	0.9
Education	16.4 (2.6)	16.2 (2.4)	0.6
*APOE* ε4 carrier %	42.0% (*n* = 45)	45.5% (*n* = 51)	0.9
MMSE score	28.1 (2.8)	28.8 (1.8)	**0.04**
CDR score	0.2 (0.3)	0.1 (0.3)	0.5
Clinical diagnosis			0.5
Cognitively Unimpaired %	76.7% (*n* = 82)	77.6% (*n* = 87)	
Clinical diagnosis of AD %	20.6% (*n* = 22)	15.2% (*n* = 17)	
Uncertain Dementia %	2.8% (*n* = 3)	6.3% (*n* = 7)	
Aβ-positive %	46.7% (*n* = 63)	53.3% (*n* = 72)	0.5

Data shown as mean (SD) for continuous variables and % (count) for categorical variables. Knight Alzheimer Disease Research Center (Knight-ADRC), Alzheimer Disease (AD), Mini Mental State Examination (MMSE), Clinical Dementia Rating (CDR), Apolipoprotein (APOE), Amyloid-beta (Aβ). Participants were classified as *APOE* ε4-positive if they carried at least one ε4 allele (genotypes 24, 34, or 44)

**Table 2:** Replicated analyses with the tau-PET entorhinal cortex as the outcome measure

Cohort	Model	Estimate	SE	*t*-value	*P*-value
BioFINDER-2 (*n* = 446)	Model A	0.70	0.04	21.22	< 0.0001
	MTBR-tau243				
Knight-ADRC (*n* = 219)	Model A				
	MTBR-tau243	0.6	0.05	11.2	< 0.0001
BioFINDER-2 Aβ + Subgroup (*n* = 302)	Model A	0.72	0.04	17.6	< 0.0001
	MTBR-tau243				
Knight-ADRC Aβ + Subgroup (*n* = 135)	Model A				
	MTBR-tau243	0.6	0.06	9.7	< 0.0001
BioFINDER-2 (*n* = 446)	Model B				
	SexF*MTBR-tau243	−0.05	0.07	−0.7	0.5
Knight-ADRC (*n* = 219)	Model B				
	SexF*MTBR-tau243	0.09	0.09	1.0	0.3
BioFINDER-2 Aβ + Subgroup (*n* = 302)	Model B				
	SexF*MTBR-tau243	−0.1	0.09	−1.5	0.1
Knight-ADRC Aβ + Subgroup (*n* = 135)	Model B				
	SexF*MTBR-tau243	0.1	0.1	0.9	0.4

Model A includes age, sex, and CSF Aβ-status as predictors of tau-PET entorhinal cortex uptake. Model B includes the same terms as Model A and adds an interaction term between sex and MTBR-tau243. Males are the reference group in all analyses. Knight Alzheimer Disease Research Center (ADRC), Amyloid-beta (Aβ), Standard Error (SE)

Supplementary analyses across additional tau-PET ROIs in the Knight-ADRC full cohort revealed that none of the associations between sex and MTBR-tau243 remained significant following FDR correction. In contrast, in the Bio-FINDER-2 full cohort, the sex by MTBR-tau243 interaction remained significant after FDR correction in the caudal anterior cingulate, frontal pole, medial orbitofrontal cortex, and rostral anterior cingulate. In these regions, males exhibited a stronger association between CSF MTBR-tau243 and tau-PET uptake. However, when analyses were restricted to the Aβ-positive subgroups in both cohorts, none of the associations between sex and MTBR-tau243 remained significant following FDR correction. Importantly, since sex differences in the association between tau-PET and MTBR-tau243 were not observed across Aβ-positive subgroups, this suggests that the stronger male associations observed in select frontal ROIs in the full BioFINDER-2 cohort are unlikely to be attributable to AD-related pathology. A whole-brain map of β coefficients is shown in Supplementary Figs. 1, 2, 3, and 4, and detailed results are provided in Supplementary Tables 1 and 2. Sex-stratified slope estimates for the association between MTBR-tau243 and tau-PET uptake were as follows: in the BioFINDER-2 full cohort, β = 0.747 for females (SE = 0.0677, 95% CI: 0.669–0.897) and β = 0.783 for males (SE = 0.0577, 95% CI: 0.669–0.897); in the Knight-ADRC full cohort, β = 0.685 for females (SE = 0.0579, 95% CI: 0.561–0.789) and β = 0.675 for males (SE = 0.0705, 95% CI: 0.546–0.823); in the BioFINDER-2 Aβ-positive subgroup, β = 0.801 for females (SE = 0.0618, 95% CI: 0.679–0.923) and β = 0.820 for males (SE = 0.0541, 95% CI: 0.713–0.927); and in the Knight-ADRC Aβ-positive subgroup, β = 0.733 for females (SE = 0.0887, 95% CI: 0.558–0.909) and β = 0.677 for males (SE = 0.0740, 95% CI: 0.531–0.824) (Supplementary Table 3).

## Discussion

The present study examined whether sex moderated the association between CSF MTBR-tau243 and tau-PET in two independent cohorts, with the goal of clarifying the extent to which previously observed sex differences in tau-PET generalize to other modalities. Across cohorts, we observed significant associations between CSF MTBR-tau243 and tau-PET, consistent with prior research [33]. In both cohorts, sex did not moderate the relationship between CSF MTBR-tau243 and tau-PET, two distinct measures of aggregated tau pathology. This finding was consistent in both Aβ-positive subgroups, groups at heightened risk for tau pathology. Supplementary analyses extending the Aβ-positive subgroup analyses across additional tau-PET ROIs likewise revealed no significant sex by MTBR-tau243 interactions.

Our findings indicate that sex differences in tau-PET signal [11–18] are not attributable to methodological effects, but reflect a true biological susceptibility. Notably, the comparison of tau-PET and CSF MTBR-tau243, in two independent cohorts, provides novel converging evidence from two distinct measures of aggregated tau. This interpretation is further reinforced by CSF studies [8, 50–53] and autopsy investigations [19–23] demonstrating greater tau burden in females. The consistency of this pattern across multiple modalities supports the conclusion that increased female susceptibility to tau represents a genuine biological effect. In BioFINDER-2, females exhibited greater tau burden than males measured by both tau-PET and CSF MTBR-tau243 in the full cohort and within the Aβ-positive subgroup. These sex differences were not observed in the Knight-ADRC. This discrepancy is likely attributable to greater statistical power in BioFINDER-2 and the inclusion of a higher proportion of cognitively impaired participants. Importantly, prior work within the larger Knight-ADRC cohort has demonstrated greater tau-PET burden in females [54], further suggesting that the absence of a significant effect here may reflect limited power.

The confirmation of actual sex differences in tau pathology provided here is biologically informative for interpreting sex effects in AD prevalence and in the relationship between pathological burden and cognition. Prior work suggests that females exhibit greater cognitive resilience at initial levels of AD pathology but experience more accelerated cognitive decline as the disease progresses [14, 55–57], highlighting the existence and complexity of sex-specific disease trajectories. These distinctions are particularly relevant in the context of therapeutic development. Given evidence that tau burden predicts treatment response [24], along with emerging data potentially hinting at sex differences in the efficacy of currently approved amyloid targeting therapies [25, 26], accurately characterizing sex differences in tau pathology is essential for understanding potential sources of heterogeneity in clinical trial outcomes. Demonstrating that greater vulnerability to tau pathology in females is not attributable to methodological effects, but reflects a true biological susceptibility, provides a stronger biological foundation for incorporating sex into therapeutic stratification, clinical trial design, and precision-medicine approaches.

Strengths of our study include the use of two well-characterized cohorts with two distinct tau-PET tracers and the incorporation of MTBR-tau243, a novel biomarker of aggregated tau pathology. The main limitation is that the Knight-ADRC and BioFINDER-2 cohorts predominantly consist of highly educated, high socioeconomic status, non-Hispanic white individuals, which limits the generalizability of our findings to diverse populations. Future work should examine whether sex moderates the association between CSF MTBR-tau243 and tau-PET in larger, more diverse populations.

## Conclusions

The present study demonstrated that the associations between tau-PET and MTBR-tau-243 did not differ by sex. Thus, sex differences in tau-PET signal are unlikely explained by differences in tracer binding but rather reflect a biological susceptibility to tau pathology in females, compared to males. This advances the field’s understanding of sex as a critical biological variable that influences tau pathology in AD and underscores the need for sex-specific approaches in research and therapeutic development.

## Supplementary Information

Below is the link to the electronic supplementary material.

Supplementary file1 (DOCX 947 KB)

## Data Availability

All data supporting the findings of this study are available upon request from the corresponding author. The data are not publicly available due to privacy or ethical restrictions.
