# Supplementary material for: Tau-PET and CSF MTBR-tau243 comparisons validate increased tau aggregation in females
Source: Eur J Nucl Med Mol Imaging. 2026 May 27;53(10):5690–8. doi: 10.1007/s00259-026-07934-y (PMC13421202; doi:10.1007/s00259-026-07934-y)
Supplement: Supplementary file 1 — Supplementary file1 (DOCX 947 KB) [file 259_2026_7934_MOESM1_ESM.docx]

**Supplementary**

**Tau-PET and CSF MTBR-tau243 comparisons validate increased tau aggregation in females**

Carling G. Robinson^1*^, Alexa Pichet Binette^6,7,8*^, Kanta Horie^3,4,5^, Chihiro Sato^3,4^, Suzanne E. Schindler^3^, Randall J. Bateman^3,4^, Tammie L. S. Benzinger^2^, Shorena Janelidze^8^, Ellen Singleton^8^, Erik Stomrud^8,12^, Gordon Zhaoqi An^2^, Taylor J. Pedersen^1^, Sebastian Palmqvist^8,12^, Niklas Mattsson-Carlgren^8,12^, John C. Morris^3^, Oskar Hansson^8^, Brian A. Gordon^2*^, Rik Ossenkoppele^8,9,10*^

^1^Department of Psychological and Brain Sciences, Washington University in St. Louis, MO, USA

^2^Department of Radiology, Washington University in St. Louis, MO, USA

^3^Department of Neurology, Washington University in St. Louis, MO, USA

^4^Tracy Family SILQ Center, Washington University School of Medicine, St. Louis, MO, USA

^5^Eisai Inc, Nutley, NJ, USA

^6^Department of Physiology and Pharmacology, Université de Montréal, Montréal, Quebec, Canada

^7^Canada Centre de Recherche de l’Institut Universitaire de Gériatrie de Montréal, Montréal, Quebec, Canada

^8^Clinical Memory Research Unit, Department of Clinical Sciences Malmö, Lund University, Lund, Sweden

^9^Alzheimer Center Amsterdam, Department of Neurology, Vrije Universiteit Amsterdam, Amsterdam UMC VUmc, Amsterdam, The Netherlands

^10^Amsterdam Neuroscience, Department of Neurodegeneration, Amsterdam, The Netherlands

^11^Memory Clinic, Skåne University Hospital, Malmö, Sweden

^12^Wallenberg Center for Molecular Medicine, Lund University, Lund, Sweden

^*^ shared first and last authorships

**Supplementary Figure 1. Brain map displaying analyses examining the sex*MTBR-tau243 on additional tau-PET ROIs in the BioFINDER-2 cohort.** Linear regression models showed that when examining the sex × MTBR-tau243 interaction model across additional tau-PET regions in the BioFINDER-2 cohort, the caudal anterior cingulate, frontal pole, medial orbitofrontal cortex, and rostral anterior cingulate regions remained significant after correcting for multiple comparisons using FDR correction. In these regions, males exhibited a stronger association between CSF MTBR-tau243 and tau-PET uptake. Age (continuous) sex (binary Male/Female), and Aβ status (binary +/-) were included as covariates in all models. Results were derived from averaged left and right hemisphere values, but are displayed on the left hemisphere for visualization. Regions examined included the banks of the superior temporal sulcus, caudal anterior cingulate, caudal middle frontal, corpus callosum, cuneus, entorhinal cortex, frontal pole, fusiform gyrus, inferior parietal cortex, inferior temporal cortex, insula, isthmus cingulate, lateral occipital cortex, lateral orbitofrontal cortex, lingual gyrus, medial orbitofrontal cortex, middle temporal cortex, paracentral cortex, parahippocampal gyrus, pars opercularis, pars orbitalis, pars triangularis, pericalcarine cortex, postcentral gyrus, posterior cingulate, precentral gyrus, precuneus, rostral anterior cingulate, rostral middle frontal cortex, superior frontal cortex, superior parietal cortex, superior temporal cortex, supramarginal gyrus, temporal pole, and transverse temporal cortex.

**
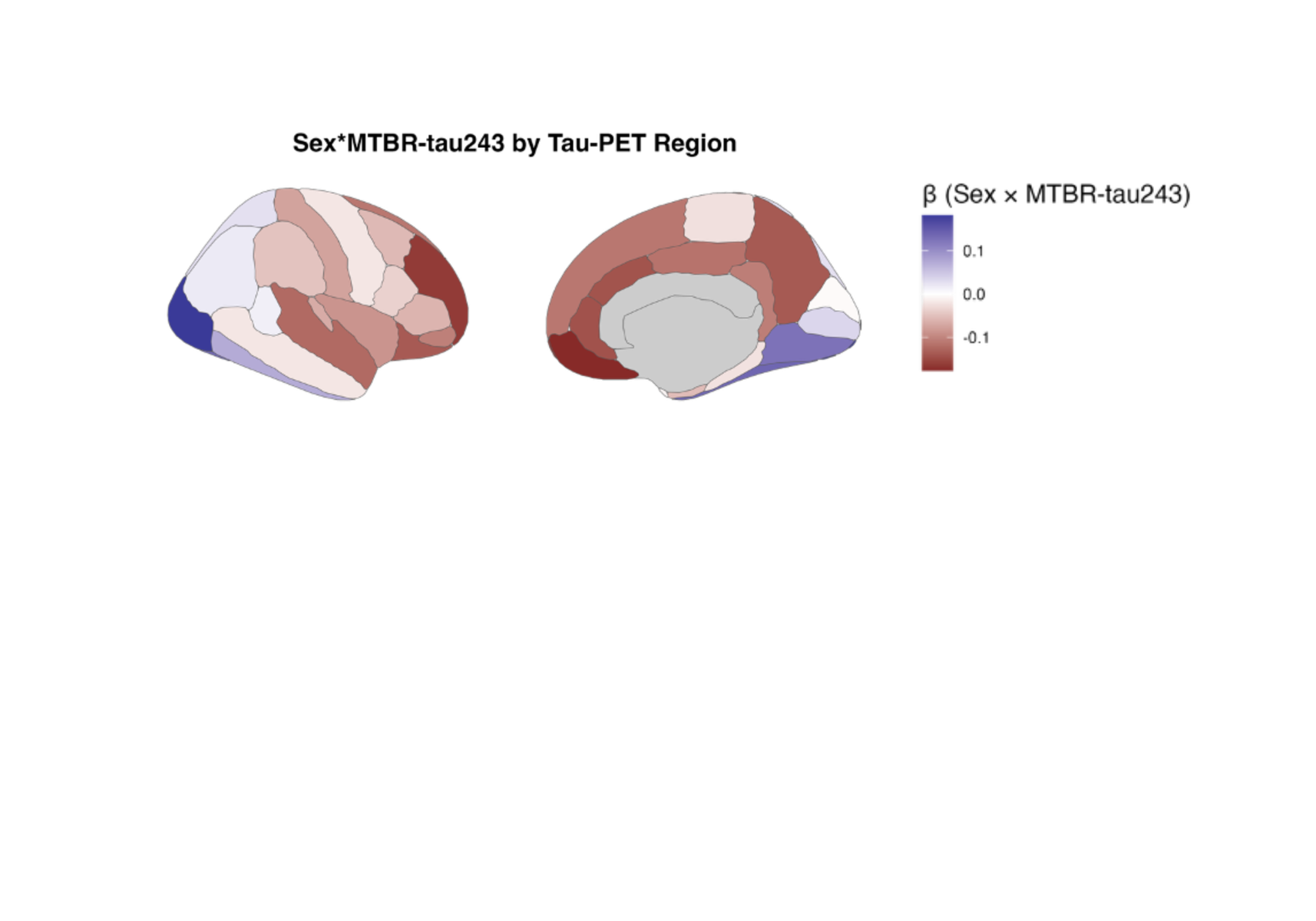
**

**Supplementary Figure 2. Brain map displaying analyses examining the sex*MTBR-tau243 on additional tau-PET ROIs in the Knight-ADRC cohort.** Linear regression models showed that when examining the sex × MTBR-tau243 interaction model across additional tau-PET regions in the Knight-ADRC cohort, none of the regions remained significant after correcting for multiple comparisons using FDR correction. Age (continuous) sex (binary Male/Female), and Aβ status (binary +/-) were included as covariates in all models. Results were derived from averaged left and right hemisphere values, but are displayed on the left hemisphere for visualization. Regions examined included the banks of the superior temporal sulcus, caudal anterior cingulate, caudal middle frontal, corpus callosum, cuneus, entorhinal cortex, frontal pole, fusiform gyrus, inferior parietal cortex, inferior temporal cortex, insula, isthmus cingulate, lateral occipital cortex, lateral orbitofrontal cortex, lingual gyrus, medial orbitofrontal cortex, middle temporal cortex, paracentral cortex, parahippocampal gyrus, pars opercularis, pars orbitalis, pars triangularis, pericalcarine cortex, postcentral gyrus, posterior cingulate, precentral gyrus, precuneus, rostral anterior cingulate, rostral middle frontal cortex, superior frontal cortex, superior parietal cortex, superior temporal cortex, supramarginal gyrus, temporal pole, and transverse temporal cortex.

**
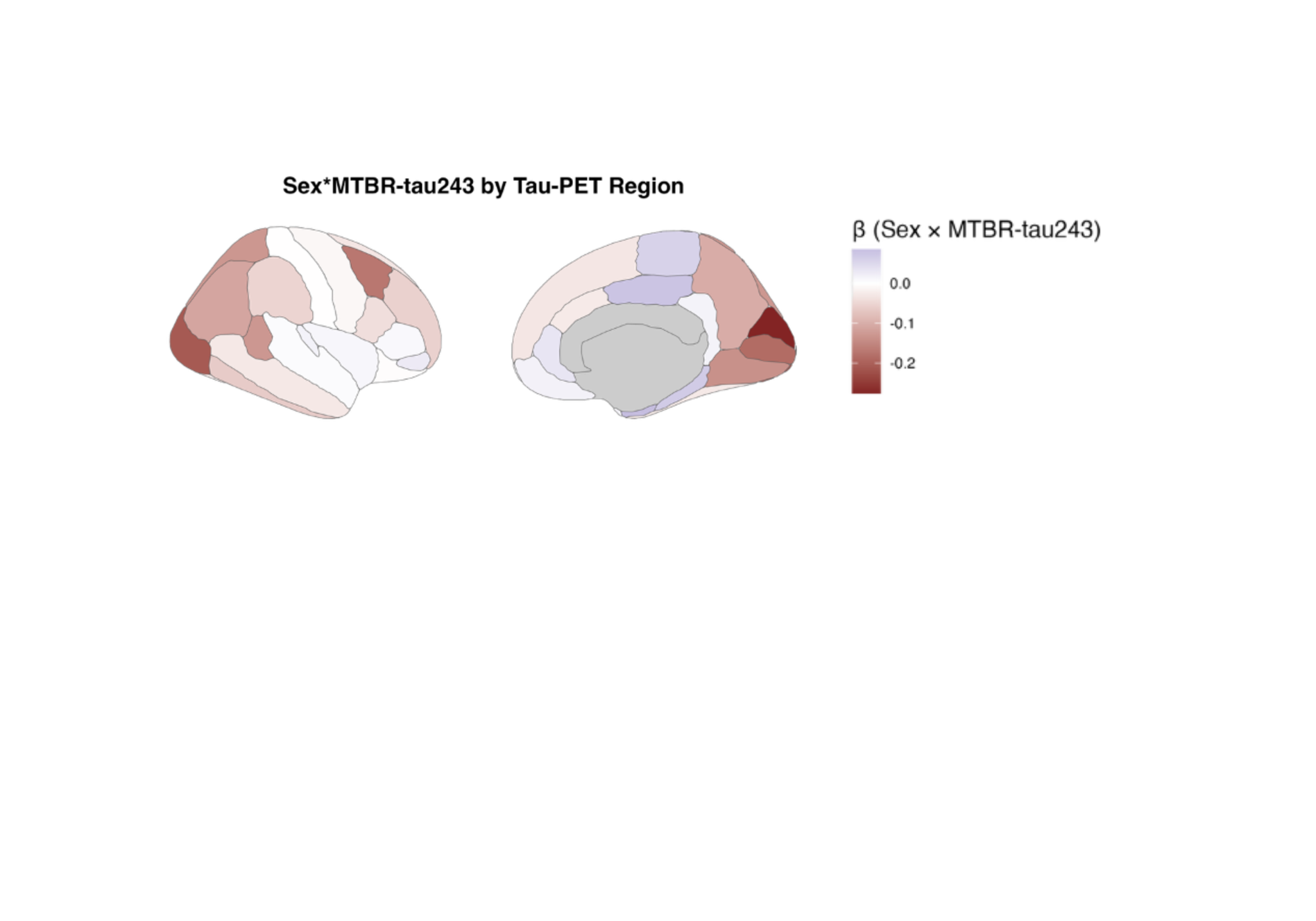
**

**Supplementary Table 1. Displays Betas, unadjusted p-values, and FDR adjusted p-values for analyses examining the sex*MTBR-tau243 on additional tau-PET ROIs in the BioFINDER-2 and Knight-ADRC cohorts.** Age (continuous), sex (binary Male/Female) and Aβ status (binary +/-) were included as covariates in all models. Results were corrected for multiple comparison using the FDR threshold of *P* < 0.05.

|  | **BioFINDER-2** | | | **Knight-ADRC** | | |
| --- | --- | --- | --- | --- | --- | --- |
| **Region** | **Beta** | ***P* (raw)** | ***P* (FDR)** | **Beta** | ***P* (raw)** | ***P* (FDR)** |
| Medial Orbitofrontal | -0.177 | 0.000 | **0.010** | 0.0180 | 0.777 | 0.995 |
| Caudal Anterior Cingulate | -0.142 | 0.001 | **0.017** | -0.0256 | 0.657 | 0.995 |
| Frontal Pole | -0.165 | 0.001 | **0.017** | 0.0076 | 0.910 | 0.995 |
| Rostral Anterior Cingulate | -0.144 | 0.003 | **0.031** | 0.0332 | 0.582 | 0.995 |
| Lateral Orbitofrontal | -0.137 | 0.010 | 0.089 | -0.0031 | 0.960 | 0.995 |
| Accumbens | -0.097 | 0.013 | 0.093 | 0.0355 | 0.685 | 0.995 |
| Rostral Middle Frontal | -0.163 | 0.022 | 0.137 | -0.0563 | 0.490 | 0.995 |
| Insula | -0.087 | 0.037 | 0.204 | 0.0119 | 0.835 | 0.995 |
| Pars orbitalis | -0.104 | 0.056 | 0.243 | 0.0266 | 0.670 | 0.995 |
| Postcentral | -0.075 | 0.066 | 0.243 | -0.0009 | 0.989 | 0.995 |
| Superior Frontal | -0.112 | 0.050 | 0.243 | -0.0299 | 0.657 | 0.995 |
| Superior Temporal | -0.123 | 0.066 | 0.243 | 0.0052 | 0.944 | 0.995 |
| Lingual | 0.125 | 0.081 | 0.255 | -0.1374 | 0.091 | 0.995 |
| Posterior Cingulate | -0.115 | 0.077 | 0.255 | 0.0767 | 0.338 | 0.995 |
| Lateral Occipital | 0.181 | 0.089 | 0.261 | -0.2090 | 0.093 | 0.995 |
| Transverse Temporal | -0.068 | 0.103 | 0.283 | 0.0170 | 0.782 | 0.995 |
| Fusiform | 0.137 | 0.137 | 0.356 | -0.0265 | 0.789 | 0.995 |
| Precuneus | -0.137 | 0.157 | 0.384 | -0.1020 | 0.419 | 0.995 |
| Isthmus Cingulate | -0.106 | 0.190 | 0.441 | 0.0158 | 0.872 | 0.995 |
| Pars triangularis | -0.060 | 0.231 | 0.509 | 0.0086 | 0.892 | 0.995 |
| Pericalcarine | 0.034 | 0.412 | 0.856 | -0.1858 | 0.014 | 0.305 |
| Amygdala | -0.050 | 0.515 | 0.856 | 0.0197 | 0.861 | 0.995 |
| Caudal Middle Frontal | -0.056 | 0.507 | 0.856 | -0.1708 | 0.120 | 0.995 |
| Entorhinal | -0.054 | 0.439 | 0.856 | 0.0857 | 0.340 | 0.995 |
| Inferior Temporal | 0.072 | 0.467 | 0.856 | -0.0631 | 0.607 | 0.995 |
| Paracentral | -0.024 | 0.525 | 0.856 | 0.0586 | 0.353 | 0.995 |
| Pars opercularis | -0.037 | 0.525 | 0.856 | -0.0399 | 0.582 | 0.995 |
| Supramarginal | -0.048 | 0.550 | 0.864 | -0.0523 | 0.604 | 0.995 |
| Caudate | 0.014 | 0.666 | 0.923 | -0.0162 | 0.847 | 0.995 |
| Choroid Plexus | 0.023 | 0.623 | 0.923 | -0.0223 | 0.856 | 0.995 |
| Hippocampus | 0.015 | 0.734 | 0.923 | -0.0006 | 0.995 | 0.995 |
| Pallidum | 0.017 | 0.723 | 0.923 | -0.0669 | 0.588 | 0.995 |
| Parahippocampal | -0.023 | 0.673 | 0.923 | 0.0674 | 0.305 | 0.995 |
| Precentral | -0.020 | 0.663 | 0.923 | -0.0101 | 0.871 | 0.995 |
| Thalamus Proper | 0.010 | 0.694 | 0.923 | -0.0475 | 0.508 | 0.995 |
| Brainstem | 0.006 | 0.835 | 0.932 | -0.0137 | 0.791 | 0.995 |
| Inferior Parietal | 0.018 | 0.868 | 0.932 | -0.1110 | 0.416 | 0.995 |
| Middle Temporal | -0.020 | 0.831 | 0.932 | -0.0276 | 0.814 | 0.995 |
| Putamen | 0.007 | 0.852 | 0.932 | -0.1132 | 0.251 | 0.995 |
| Superior Parietal | 0.026 | 0.776 | 0.932 | -0.1296 | 0.276 | 0.995 |
| Temporal Pole | -0.011 | 0.869 | 0.932 | 0.0169 | 0.824 | 0.995 |
| Banks of the Superior Temporal Sulcus | 0.014 | 0.907 | 0.950 | -0.1288 | 0.356 | 0.995 |
| Cuneus | -0.004 | 0.956 | 0.956 | -0.2768 | 0.005 | 0.206 |
| Ventral Diencephalon | -0.002 | 0.955 | 0.956 | -0.0384 | 0.608 | 0.995 |

Note: Microtubule binding region tau species containing residue 243 (MTBR-tau243), Positron Emission Tomography (PET), Knight Alzheimer Disease Research Center (ADRC), Region of Interest (ROI), Amyloid-beta (Aβ), False Discovery Rate (FDR).

**Supplementary Figure 3. Brain map displaying analyses examining the sex*MTBR-tau243 on additional tau-PET ROIs in the BioFINDER-2 Aβ+ subgroup.** Linear regression models showed that when examining the sex × MTBR-tau243 interaction model across additional tau-PET regions in the BioFINDER-2 Aβ+ subgroup, none of the regions remained significant after correcting for multiple comparisons using FDR correction. Age (continuous) and sex (binary Male/Female) were included as covariates in all models. Results were derived from averaged left and right hemisphere values, but are displayed on the left hemisphere for visualization. Regions examined included the banks of the superior temporal sulcus, caudal anterior cingulate, caudal middle frontal, corpus callosum, cuneus, entorhinal cortex, frontal pole, fusiform gyrus, inferior parietal cortex, inferior temporal cortex, insula, isthmus cingulate, lateral occipital cortex, lateral orbitofrontal cortex, lingual gyrus, medial orbitofrontal cortex, middle temporal cortex, paracentral cortex, parahippocampal gyrus, pars opercularis, pars orbitalis, pars triangularis, pericalcarine cortex, postcentral gyrus, posterior cingulate, precentral gyrus, precuneus, rostral anterior cingulate, rostral middle frontal cortex, superior frontal cortex, superior parietal cortex, superior temporal cortex, supramarginal gyrus, temporal pole, and transverse temporal cortex.

**
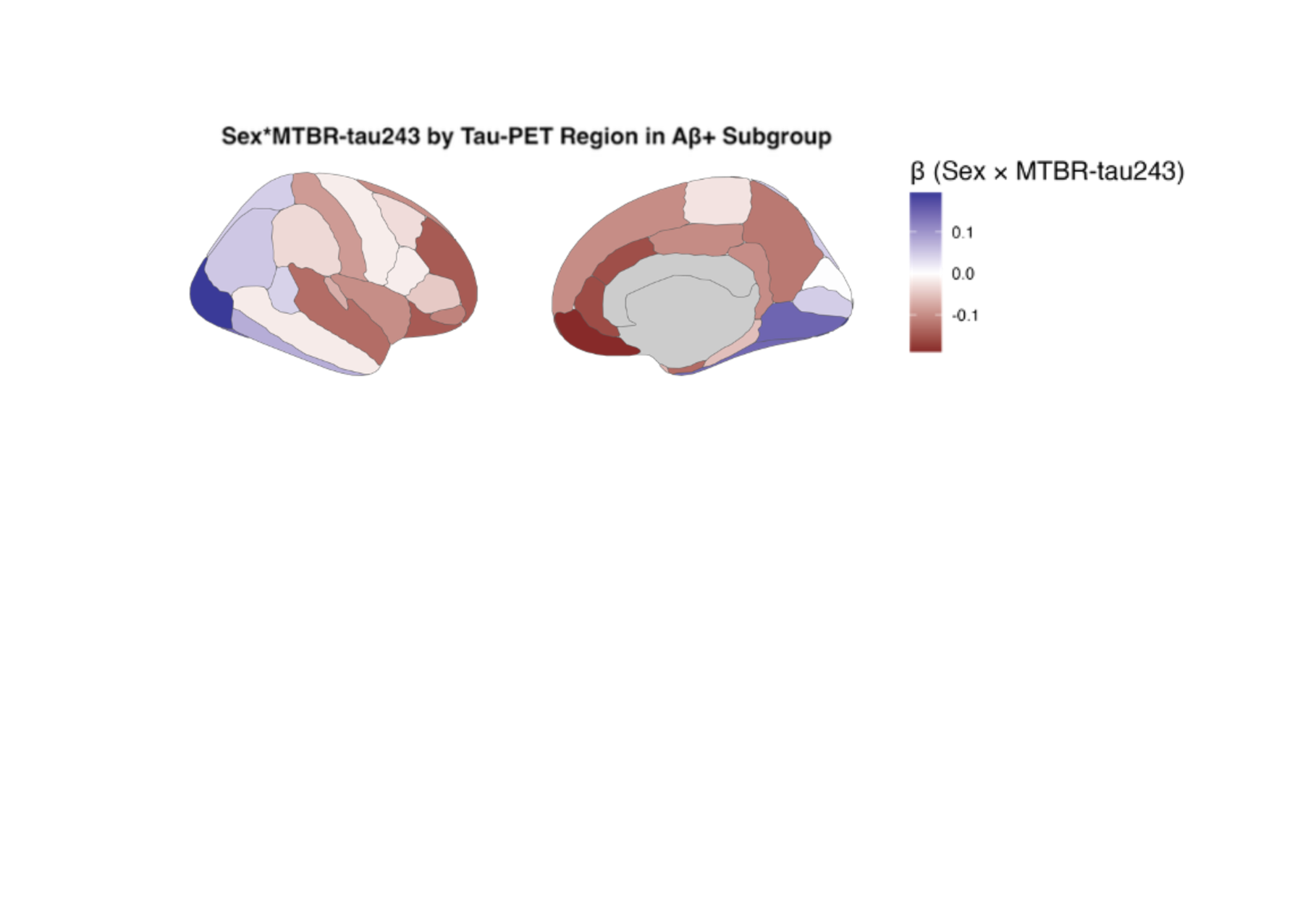
**

**Supplementary Figure 4. Brain map displaying analyses examining the sex*MTBR-tau243 on additional tau-PET ROIs in the Knight-ADRC Aβ+ subgroup.** Linear regression models showed that when examining the sex × MTBR-tau243 interaction model across additional tau-PET regions in the Knight-ADRC Aβ+ subgroup, none of the regions remained significant after correcting for multiple comparisons using FDR correction. Age (continuous) and sex (binary Male/Female) were included as covariates in all models. Results were derived from averaged left and right hemisphere values, but are displayed on the left hemisphere for visualization. Regions examined included the banks of the superior temporal sulcus, caudal anterior cingulate, caudal middle frontal, corpus callosum, cuneus, entorhinal cortex, frontal pole, fusiform gyrus, inferior parietal cortex, inferior temporal cortex, insula, isthmus cingulate, lateral occipital cortex, lateral orbitofrontal cortex, lingual gyrus, medial orbitofrontal cortex, middle temporal cortex, paracentral cortex, parahippocampal gyrus, pars opercularis, pars orbitalis, pars triangularis, pericalcarine cortex, postcentral gyrus, posterior cingulate, precentral gyrus, precuneus, rostral anterior cingulate, rostral middle frontal cortex, superior frontal cortex, superior parietal cortex, superior temporal cortex, supramarginal gyrus, temporal pole, and transverse temporal cortex.


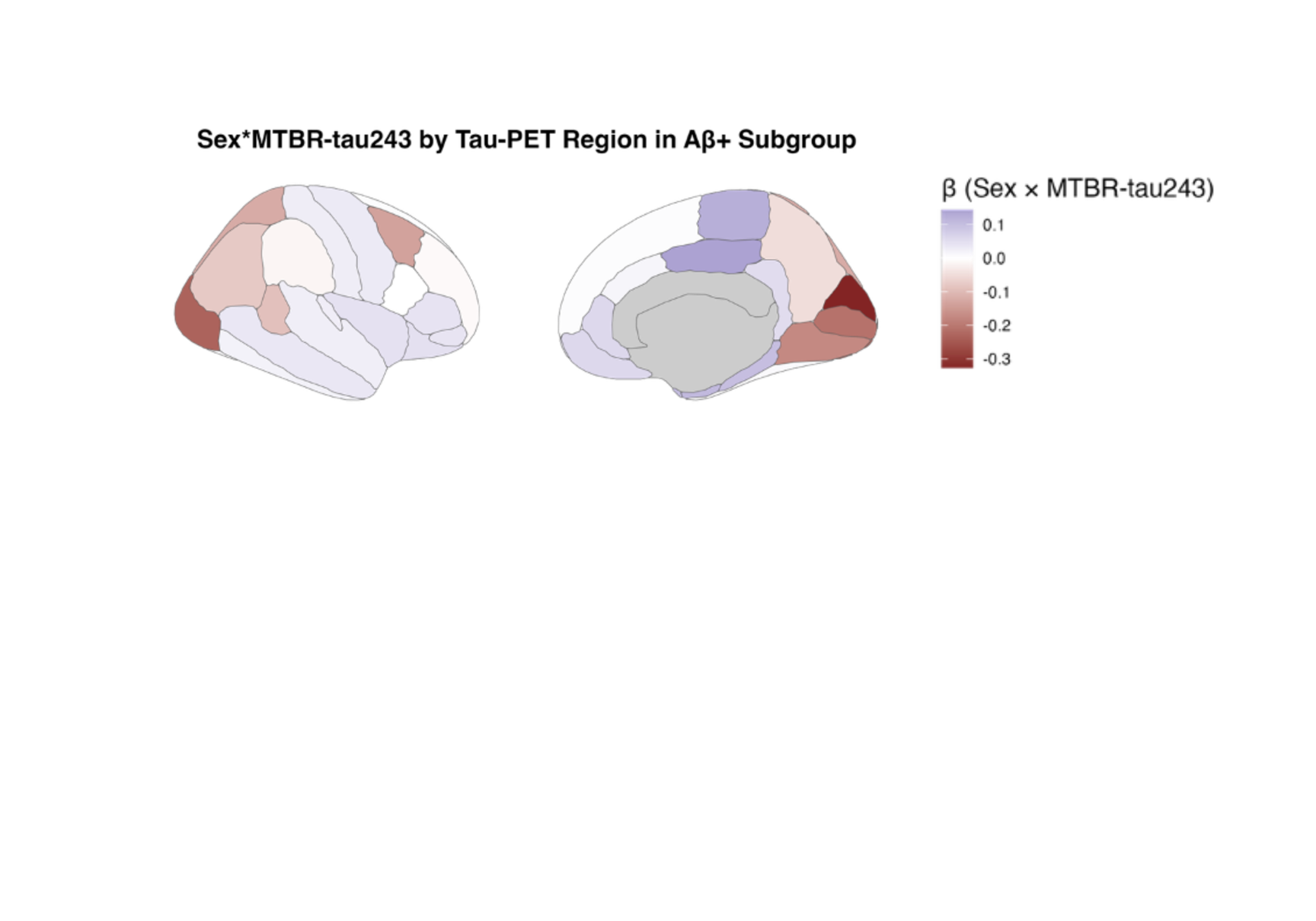


**Supplementary Table 2. Displays Betas, unadjusted p-values, and FDR adjusted p-values for analyses examining the sex*MTBR-tau243 on additional tau-PET ROIs in the BioFINDER-2 and Knight-ADRC Aβ+ subgroups.** Age (continuous) and sex (binary Male/Female) were included as covariates in all models. Results were corrected for multiple comparison using the FDR threshold of *P* < 0.05.

|  | **BioFINDER-2 Aβ+ subgroup** | | | **Knight-ADRC Aβ+ subgroup** | | |
| --- | --- | --- | --- | --- | --- | --- |
| **Region** | **Beta** | ***P* (raw)** | ***P* (FDR)** | **Beta** | ***P* (raw)** | ***P* (FDR)** |
| Accumbens | -0.122 | 0.011 | 0.113 | 0.1216 | 0.209 | 0.952 |
| Caudal Anterior Cingulate | -0.157 | 0.006 | 0.113 | 0.0151 | 0.819 | 0.952 |
| Frontal Pole | -0.158 | 0.013 | 0.113 | 0.0366 | 0.681 | 0.952 |
| Medial Orbitofrontal | -0.190 | 0.003 | 0.113 | 0.0592 | 0.432 | 0.952 |
| Rostral Anterior Cingulate | -0.160 | 0.013 | 0.113 | 0.0592 | 0.391 | 0.952 |
| Lateral Orbitofrontal | -0.146 | 0.035 | 0.258 | 0.0462 | 0.524 | 0.952 |
| Insula | -0.099 | 0.072 | 0.452 | 0.0454 | 0.481 | 0.952 |
| Lingual | 0.148 | 0.115 | 0.496 | -0.1736 | 0.129 | 0.952 |
| Pars orbitalis | -0.109 | 0.124 | 0.496 | 0.0402 | 0.597 | 0.952 |
| Postcentral | -0.087 | 0.096 | 0.496 | 0.0275 | 0.728 | 0.952 |
| Rostral Middle Frontal | -0.146 | 0.113 | 0.496 | -0.0078 | 0.943 | 0.977 |
| Amygdala | -0.125 | 0.193 | 0.499 | 0.0632 | 0.670 | 0.952 |
| Entorhinal | -0.130 | 0.139 | 0.499 | 0.1077 | 0.375 | 0.952 |
| Lateral Occipital | 0.195 | 0.167 | 0.499 | -0.2322 | 0.196 | 0.952 |
| Superior Frontal | -0.100 | 0.177 | 0.499 | 0.0048 | 0.955 | 0.977 |
| Superior Temporal | -0.129 | 0.148 | 0.499 | 0.0254 | 0.798 | 0.952 |
| Transverse Temporal | -0.070 | 0.191 | 0.499 | 0.0264 | 0.727 | 0.952 |
| Fusiform | 0.146 | 0.237 | 0.549 | 0.0086 | 0.951 | 0.977 |
| Posterior Cingulate | -0.101 | 0.227 | 0.549 | 0.1442 | 0.179 | 0.952 |
| Isthmus Cingulate | -0.100 | 0.342 | 0.705 | 0.0526 | 0.699 | 0.952 |
| Precuneus | -0.118 | 0.341 | 0.705 | -0.0488 | 0.786 | 0.952 |
| Ventral Diencephalon | -0.039 | 0.353 | 0.705 | -0.0335 | 0.678 | 0.952 |
| Pericalcarine | 0.046 | 0.391 | 0.748 | -0.2092 | 0.037 | 0.822 |
| Temporal Pole | -0.064 | 0.429 | 0.786 | 0.1016 | 0.310 | 0.952 |
| Parahippocampal | -0.055 | 0.448 | 0.789 | 0.0993 | 0.248 | 0.952 |
| Pars triangularis | -0.047 | 0.470 | 0.796 | 0.0418 | 0.605 | 0.952 |
| Hippocampus | -0.035 | 0.525 | 0.829 | 0.0428 | 0.683 | 0.952 |
| Putamen | -0.025 | 0.528 | 0.829 | -0.0900 | 0.430 | 0.952 |
| Inferior Temporal | 0.076 | 0.569 | 0.863 | 0.0200 | 0.907 | 0.977 |
| Paracentral | -0.024 | 0.621 | 0.911 | 0.1238 | 0.106 | 0.952 |
| Banks of the Superior Temporal Sulcus | 0.041 | 0.790 | 0.915 | -0.0907 | 0.646 | 0.952 |
| Brainstem | -0.011 | 0.709 | 0.915 | -0.0140 | 0.796 | 0.952 |
| Caudal Middle Frontal | -0.030 | 0.787 | 0.915 | -0.1355 | 0.376 | 0.952 |
| Inferior Parietal | 0.051 | 0.712 | 0.915 | -0.0783 | 0.689 | 0.952 |
| Pallidum | -0.022 | 0.658 | 0.915 | -0.0781 | 0.593 | 0.952 |
| Precentral | -0.016 | 0.791 | 0.915 | 0.0322 | 0.687 | 0.952 |
| Superior Parietal | 0.044 | 0.707 | 0.915 | -0.1222 | 0.473 | 0.952 |
| Supramarginal | -0.033 | 0.752 | 0.915 | -0.0139 | 0.921 | 0.977 |
| Caudate | -0.006 | 0.862 | 0.948 | -0.0246 | 0.802 | 0.952 |
| Pars opercularis | -0.015 | 0.841 | 0.948 | 0.0004 | 0.997 | 0.997 |
| Middle Temporal | -0.017 | 0.891 | 0.956 | 0.0364 | 0.825 | 0.952 |
| Cuneus | 0.002 | 0.986 | 0.996 | -0.3259 | 0.018 | 0.822 |
| Choroid Plexus | 0.001 | 0.986 | 0.996 | -0.0558 | 0.706 | 0.952 |
| Thalamus Proper | 0.000 | 0.996 | 0.996 | -0.0199 | 0.784 | 0.952 |

Note: Microtubule binding region tau species containing residue 243 (MTBR-tau243), Positron Emission Tomography (PET), Knight Alzheimer Disease Research Center (ADRC), Region of Interest (ROI) Amyloid-beta (Aβ), False Discovery Rate (FDR).

**Supplementary Table 3. Displays sex-stratified estimates for associations between MTBR-tau243 and the tau-PET temporal meta-ROI.** Age (continuous), sex (binary Male/Female) and Aβ status (binary +/-) were included as covariates (except for analyses in Aβ-positive subgroups). Males are the reference group.

| **Cohort** | **Model** | **Estimate** | **SE** | **CI** |
| --- | --- | --- | --- | --- |
| BioFINDER-2  (n=446) | Model  SexF*MTBR-tau243 | F: β=0.747 | 0.0677 | 0.669 – 0.897 |
|  |  | M: β=0.783 | 0.0577 | 0.669 – 0.897 |
| Knight-ADRC  (n=219) | Model  SexF*MTBR-tau243 | F: β=0.685 | 0.0579 | 0.561– 0.789 |
|  |  | M: β=0.675 | 0.0705 | 0.546 – 0.823 |
| BioFINDER-2 Aβ+ Subgroup  (n=302) | Model  SexF*MTBR-tau243 | F: β=0.801 | 0.0618 | 0.679 - 0.923 |
|  |  | M: β=0.820 | 0.0541 | 0.713 - 0.927 |
| Knight-ADRC Aβ+ Subgroup  (n=135) | Model  SexF*MTBR-tau243 | F: β=0.733 | 0.0887 | 0.558 – 0.909 |
|  |  | M: β=0.677 | 0.0740 | 0.531 – 0.824 |

Note: Microtubule binding region tau species containing residue 243 (MTBR-tau243), Positron Emission Tomography (PET), Knight Alzheimer Disease Research Center (ADRC), Region of Interest (ROI) Amyloid-beta (Aβ), Standard Error (SE), Confidence Interval (CI), Female (F), Male (M).
